# The physicAl aCtivity Counselling for young adult cancEr SurvivorS (ACCESS) trial: A protocol for a parallel, two-arm pilot randomized controlled trial

**DOI:** 10.1371/journal.pone.0273045

**Published:** 2022-12-30

**Authors:** Jennifer Brunet, Jenson Price, Amirrtha Srikanthan, Fiona Gillison, Martyn Standage, Monica Taljaard, Mark R. Beauchamp, Jennifer Reed, Amanda Wurz

**Affiliations:** 1 School of Human Kinetics, University of Ottawa, Ottawa, Ontario, Canada; 2 Ottawa Hospital Research Institute, Ottawa, Ontario, Canada; 3 Institut du savoir Montfort, Ottawa, Ontario, Canada; 4 Department of Medicine, University of Ottawa, The Ottawa Hospital, Ottawa, Ontario, Canada; 5 Centre for Motivation and Health Behaviour Change, University of Bath, Bath, United Kingdom; 6 School of Kinesiology, University of British Columbia, Vancouver, British Columbia, Canada; 7 University of Ottawa Heart Institute, Ottawa, Ontario, Canada; 8 School of Epidemiology and Public Health, Faculty of Medicine, University of Ottawa, Ottawa, Ontario, Canada; 9 School of Kinesiology, University of the Fraser Valley, Chilliwack, British Columbia, Canada; Public Library of Science, UNITED KINGDOM

## Abstract

**Background:**

Young adults aged 18–39 years commonly experience persistent side effects following cancer treatment that can impair their quality of life. Physical activity (PA) holds promise as a behavioral intervention to mitigate persistent side effects and improve quality of life. Yet, few young adults are active enough to incur these benefits and efforts to promote PA after cancer treatment ends are lacking. Therefore, we developed a novel theory-driven behavior change intervention to promote PA via videoconferencing technology in young adults who have completed cancer treatment, and are undertaking a pilot randomized controlled trial (RCT) to gather evidence to inform the design of a large, full-scale RCT. The specific aims of this parallel, two-arm pilot RCT are to: (1) assess intervention and trial protocol feasibility and acceptability; and (2) generate data on PA behavior. To promote transparency, improve reproducibility, and serve as a reference for forthcoming publication of results, we present the study protocol for this pilot RCT (version 7) within this paper.

**Methods:**

Young adults who have completed cancer treatment are being recruited from across Canada. After informed consent is obtained and baseline assessments are completed, participants are randomized to the intervention group (i.e., a 12-week behavior change intervention delivered via videoconferencing technology by trained PA counsellors) or usual care group (i.e., no intervention). Several feasibility outcomes covering enrollment, allocation, follow-up, and analysis are tracked by study staff. Acceptability is assessed through interviews exploring participants’ experiences, thoughts, and perspectives of the trial protocol (i.e., intervention and usual care groups), as well as participants’ views of the intervention and its mode of delivery (i.e., intervention group only) and PA counsellors’ experiences delivering the intervention. PA behavior is measured using accelerometers at baseline (pre-randomization), post-intervention, and at follow-up (24 weeks post-baseline).

**Discussion:**

There are growing calls to develop interventions to support young adults’ motivation to engage in PA and adopt an active lifestyle to improve their quality of life after cancer treatment ends. Real-time videoconferencing shows promise for disseminating behavior change interventions to young adults and addressing participation barriers. Considering the importance of establishing intervention and trial protocol feasibility and acceptability prior to evaluating intervention efficacy (or effectiveness), this pilot RCT is critical to understand how participants embrace, engage with, and complete the intervention and trial protocol. Indeed, these data will help to determine which refinements, if any, are required to the intervention and trial protocol (e.g., implementation approach, evaluation methods) prior to a large, full-scale RCT aiming to test the effects of the intervention on PA behavior. Additionally, the PA behavior data collected will be useful to inform the sample size calculation for a large, full-scale RCT.

**Trial registration:**

The trial was registered with the ClinicalTrials.gov database (ID: NCT04163042) on November 14, 2019, prior to the start of the trial in February, 2021.

## Introduction

### Background and rationale

Whilst cancer remains a leading cause of death in young adults aged 18–39 years, the overall 5–year survival rate in high income countries continues to improve and now exceeds 80% [1]. Nevertheless, many young adults report persistent and disabling side effects, which can not only impair their quality of life, but can result in morbidity and early mortality [2, 3]. Young adults with a history of cancer have a heightened risk for chronic diseases (e.g., cardiovascular diseases; [3]) and are at a 10–fold increased risk for death compared to matched noncancer peers [2]. The pathways through which cancer increases the risk of morbidity and early mortality are complex and multifactorial [4]. One proposed mechanism is a lack of physical activity (PA; [4]), with several studies showing that PA is associated with improved survival outcomes [5]. Yet, most young adults do not meet levels of PA that are recommended for persons diagnosed with cancer [6]. Moreover, levels of PA remain significantly lower among young adults across the cancer continuum in comparison to matched noncancer peers [7, 8]. Disseminating interventions to increase PA among young adults after cancer treatment ends may help to reduce the burden of disease in this population.

#### Theory-based behavior change interventions

The theoretical basis of an intervention and the integration of behavior change techniques (BCTs) are characteristics that can influence intervention efficacy [9]. Theories help to identify intervention targets and outcomes because they offer explanations of complex phenomena, include key drivers of behavior change, and identify constructs to assess [10]. Self-determination theory (SDT; [11]) is a macro theory of human motivation widely used to develop behavior change interventions due to its focus on psychological factors and processes that determine motivated behavior in diverse health contexts and populations [12–14]. Within SDT, satisfying one’s psychological needs for competence, autonomy, and relatedness is theorized to influence behavioral outcomes by promoting autonomous motivation (i.e., motivation reflecting self-endorsed reasons for engaging in a behavior; [11]). Systematic reviews and empirical studies support the use of SDT to develop PA behavior change interventions for adults with and without cancer [13, 15–18]. Furthermore, within SDT, it is theorized that the extent to which one’s psychological needs are satisfied is largely influenced by their social environment [11]. Thus, the integration of behaviors and communicative techniques that social agents (e.g., PA counsellors) can use to support, or actively satisfy, participants’ psychological needs to promote autonomous motivation and behavior change are central to SDT-based behavior change interventions.

SDT-informed (i.e., those vaguely describing SDT use) and SDT-driven (i.e., those integrating SDT throughout intervention planning, design, and evaluation) behavior change interventions (hereinafter collectively referred to as SDT-based or theory-based when nonspecific to SDT) have comprised several behavioral and communicative techniques designed to support participants’ psychological needs, and in turn promote autonomous motivation and behavior change. For instance, autonomy supportive techniques such as cultivating autonomy support, providing structure, and establishing strong interpersonal relationships with participants have been used and found to promote behavior change via psychological needs satisfaction and increased autonomous motivation [19–21]. Owing to the growing use of various techniques across different SDT-based interventions, recent work has sought to identify, describe, and organize the essential techniques, referred to as either behavior change techniques (BCTs) or motivation and behavior change techniques (MBCTs), used to promote sustained behavior change, and thus critical to intervention success [22, 23]. BCTs and MBCTs are replicable components of an intervention designed to facilitate behavior change by either augmenting factors that facilitate behavior change or by mitigating factors that inhibit behavior change. This work has revealed a large range of potential BCTs/MBCTs that could be selected for SDT-based behavior change interventions [22, 23]. Whilst it remains unclear which techniques or set of techniques are most efficacious for promoting autonomous motivation and behavior change via psychological needs satisfaction, studies show PA interventions integrating BCTs/MBCTs are not only effective for adults after cancer treatment ends, but are more effective than those without BCTs/MBCTs [24, 25]. There is, however, a lack of evidence for the feasibility, acceptability, and effects of interventions based on theory (SDT or otherwise) that also integrate evidence-based BCTs/MBCTs that may effectively promote PA in young adults after cancer treatment ends [26, 27]. Because not all interventions may be feasible, acceptable, and/or effective in promoting PA in young adults who have completed cancer treatment, there is some uncertainty about the value of such interventions.

#### Real-time videoconferencing interventions

To enhance public health impact, it is important that behavior change interventions not only target theoretical factors and integrate BCTs/MBCTs, but that they be readily available to the target population. Many theory-based behavior change interventions developed to promote PA in persons diagnosed with cancer have been delivered face-to-face, which can be inconvenient for participants and reach only a small proportion of the target population. It is therefore pertinent to consider alternative means of delivering theory-based behavior change interventions, investigate the extent to which these are feasible and acceptable, and assess their effects and costs. Synchronous (or real-time) videoconferencing provides an alternative means to deliver theory-based behavior change interventions to technology-adept young adults who report heavy reliance on the Internet for access to health information and who desire interactive and trustworthy content [28–31]. The use of videoconferencing technology has the potential to reach a large proportion of young adults and can address participation barriers (e.g., pandemic restrictions, travel restrictions for those living in remote locations, scheduling difficulties; [18, 32]). Moreover, videoconferencing can reduce physician burden by shifting the responsibility of promoting PA to trained PA counsellors [33, 34], which is critical as treatment and follow-up care centres have restricted infrastructure and resources, and in some cases limited expertise, to address young adults’ specific PA needs [35]. As behavior change interventions delivered via videoconferencing technology have been shown to promote health behavior change (including PA) in clinical and non-clinical populations [36–38], the World Health Organization (among other organizations) now advocates for enhanced technology use in healthcare settings [39]. On this basis, research investigating whether a novel, SDT-driven behavior change intervention that is delivered using real-time videoconferencing and integrates empirically based BCTs/MBCTs to promote PA in young adults who have completed cancer treatment, and in turn improve patient-reported outcomes, is feasible, acceptable, effective, and cost-effective is warranted.

### Purpose and study objectives

We recently developed a novel, SDT-driven behavior change intervention that integrates empirically based BCTs/MBCTs, which if shown to be effective at promoting PA behavior (primary effectiveness outcome) and patient reported outcomes (secondary effectiveness outcomes), could be brought to scale and continue to be disseminated nationally in real-time via videoconferencing technology. As a first step, a pilot RCT is currently being undertaken. The specific objectives of this ongoing pilot RCT, named the physicAl aCtivity Counselling for young adult cancEr SurvivorS (ACCESS) trial, are to: (1) assess intervention and trial protocol feasibility and acceptability from young adults’ and PA counsellors’ perspectives; and (2) generate estimates of variance in objectively measured PA to inform the sample size calculation for a future large, full-scale RCT. As extensive resources can be wasted in the absence of a carefully piloted trial, performing this pilot RCT prior to a resource intensive RCT is needed. Results will guide the design and conduct of a future large, full-scale RCT investigating the effects of the ACCESS intervention on PA and patient reported outcomes in young adults who have completed cancer treatment to reduce the burden of disease. In this manuscript, we present the protocol for this pilot RCT to facilitate appropriate assessment of the trial, promote transparency, enhance awareness of the trial, improve reproducibility, and serve as reference for forthcoming publication of trial results [40].

## Methods/design

To enhance transparency and completeness of reporting in this manuscript, this manuscript was written in accordance with the SPIRIT (Standard Protocol Items: Recommendations for Interventional Trials) guidelines ([41]; see S1 File). Forthcoming publication of trial results will be reported according to reporting standards for pilot and feasibilities studies (i.e., Consolidated Standards of Reporting Trials (CONSORT) 2010 statement for randomised pilot and feasibility trials; [42]) and the CONSORT guidelines for eHealth interventions [43].

### Trial design

**Fig 1:**
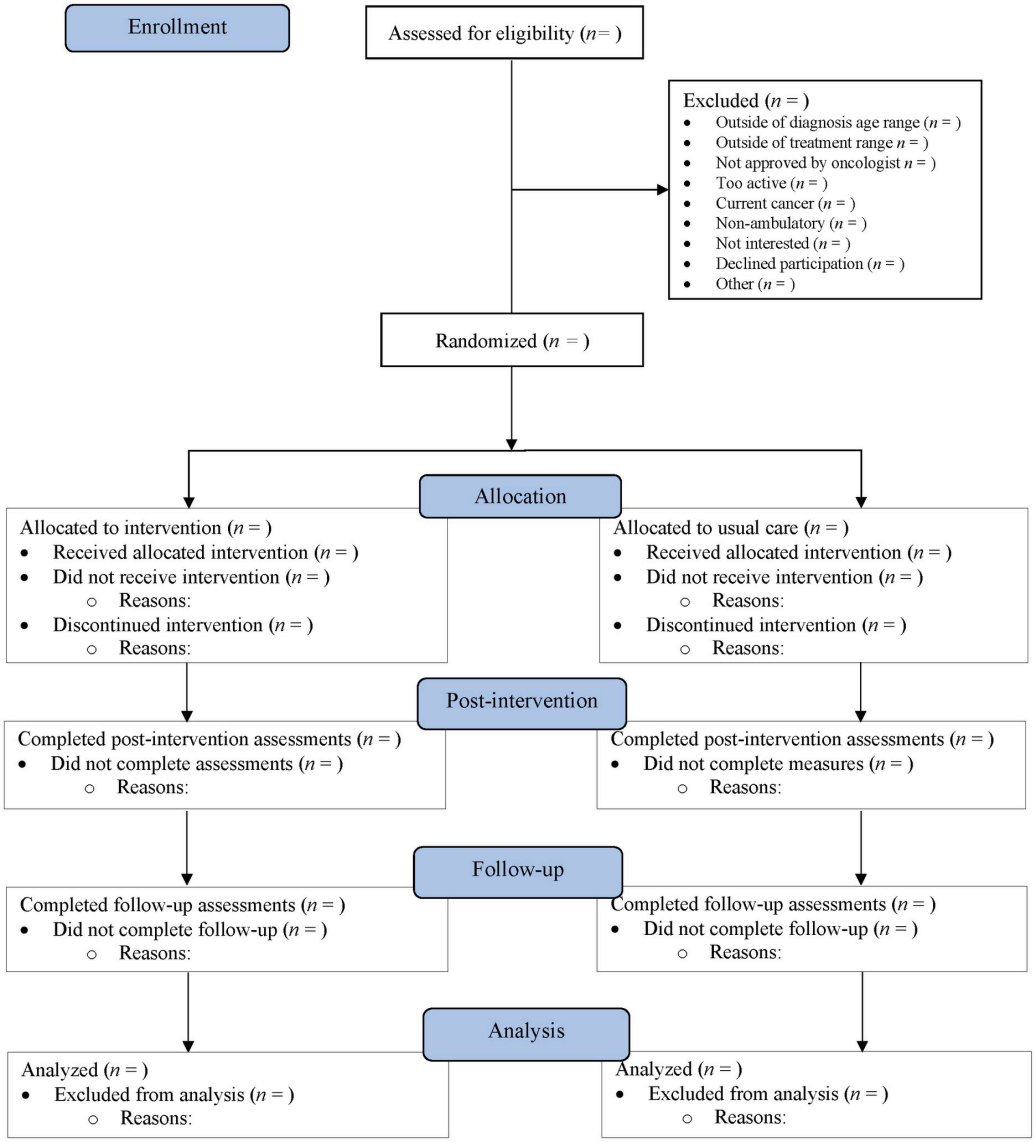
Flow of study.

The ACCESS trial is a parallel, two-arm pilot RCT with 1:1 allocation ratio, wherein quantitative and qualitative data are collected from the target population (i.e., young adults who have completed treatment for cancer; participants) and those delivering the intervention (i.e., PA counsellors). After providing informed consent digitally through a web-based form and completing baseline assessments of primary (i.e., PA behavior) and secondary (i.e., patient reported outcomes) effectiveness outcomes, young adults are randomized to one of two groups: (1) intervention group in which they receive a 12-week behavior change intervention delivered in real-time via videoconferencing technology; or (2) usual care (i.e., no intervention). Additional assessments are performed after the 12-week intervention period and at follow-up (i.e., 24 weeks post-baseline). Fig 1 summarizes the ACCESS trial design and the process of enrollment, randomization, assessments, and data analysis.

### Ethics

All participants give informed consent to participate in the ACCESS trial digitally through a web-based form housed on SurveyMonkey. As well, PA counsellors give verbal informed consent to participate in an interview at trial cessation (or when their contract ends). Ethics approval for the ACCESS trial protocol was granted by the University of Ottawa’s Office of Research Ethics and Integrity on December 12, 2020 (file no.: #H-12-19-5172) and by the Ottawa Health Science Network Research Ethics Board on January 24, 2021 (file no.: #20190643-01H). The ACCESS trial was registered with the ClinicalTrials.gov database (no.: NCT04163042) on November 14, 2019. Recruitment began in February 2021 and is ongoing. Any important protocol modifications (e.g., changes to eligibility criteria, recruitment procedures) will be reported promptly to relevant parties (e.g., research team, Institutional Review Boards, trial registry) and will be acknowledged in forthcoming publication of trial results.

All data obtained from participants (i.e., interview recordings/transcripts, questionnaire responses, accelerometer data) are considered confidential and electronic files containing personal information can only be accessed by the first author/principal investigator and study staff who sign a confidentiality form declaring that they will not disclose information that may identify a participant. A unique code is produced for each participant and used on all corresponding documentation and files to ensure anonymity. Furthermore, personal information that may enable participants to be identified are removed from interview transcripts upon transcription.

Data sharing is restricted. No data will be deposited within public data repositories as participants and PA counsellors were/are assured that their data would be kept private and confidential to the extent permitted by law, and that only the research team would have access to their data. De-identified electronic files containing anonymized quantitative and qualitative data will only be shared with the research team for analysis. All quantitative and qualitative electronic data files will be password-protected and stored on password-protected computers/laptops, a shared drive/server (i.e., University of Ottawa server), and a web-based secure and encrypted data storage service (i.e., SurveyMonkey’s Canadian Data Centre); files will be kept for at least 5 years and possibly longer. Paper materials will be stored for 5 years following the completion of the trial in locked cabinets in locked offices whose access is limited, after which point these will be destroyed securely (i.e., shredding).

### Participants, study setting, and recruitment

Young adults are recruited via two avenues: (1) locally by healthcare provider referral wherein potentially eligible young adults treated at The Ottawa Hospital (TOH) are approached by their healthcare provider to obtain consent for study staff to contact them; and (2) locally and across Canada by self-referral. For the latter, several strategies are used. First, young adults who provided permission to be contacted for research purposes through the institutional “permission to contact” process at TOH are contacted by mail and invited to participate (and thus a Data Use Agreement has been established between the University of Ottawa and TOH). Second, recruitment posters are placed in waiting rooms at TOH Cancer Centres (i.e., General Campus and the Irving Greenberg Family Cancer Centre [located at the Queensway-Carleton Hospital]), and at established Ottawa cancer survivorship centres (e.g., The Ottawa Regional Cancer Foundation, Centre for Innovation [previously known as the Ottawa Integrative Cancer Centre]). Third, advertisements are posted on the first author’s website, on relevant organizations’ websites (e.g., Young Adult Cancer Canada and Localife Ottawa), and on social media (e.g., Facebook, Twitter), and distributed to the research teams’ networks and cancer organizations’ memberships. Lastly, enrolled participants can share information about the ACCESS trial to others who they believe may be interested in participating (i.e., word of mouth). Young adults who are interested in participating are invited to contact study staff via phone or email for more information (except for those referred to study staff by healthcare providers, as in these instances, study staff contact them).

#### Eligibility criteria

Young adults are eligible to participate if they: (1) are currently between the ages of 18–39 years; (2) received a first diagnosis of invasive, non-metastatic cancer between the ages of 18–39 years; (3) completed primary treatment for cancer within the past 5 years; (4) are able to read and speak English; and (5) have access to videoconferencing technology (e.g., Zoom). Exclusion criteria include: (1) evidence of current cancer (i.e., recurrent, secondary, or relapse); (2) physical impairments precluding participation in PA; (3) self-report engaging in ≥150 minutes of moderate-to-vigorous intensity aerobic PA per week in the month prior to screening; and (4) non-ambulatory status. Young adults enrolled in other studies are potential candidates for the ACCESS trial.

### Randomization

Randomization is stratified by biological sex (male or female) to reduce a potential imbalance between groups. After baseline assessments are completed, participants are randomized to the intervention group or usual care group in a 1:1 ratio using the Clinical Trial Randomization Tool offered by the National Cancer Institute (https://ctrandomization.cancer.gov/) by a member of the first authors’ laboratory who is not involved in the who reveals group allocation to study staff, who then reveals group allocation to participants (after they completed their baseline assessments). This approach prevents study staff from knowing the sequence for new recruits or from influencing the sequence. In addition, since multiple participants can complete baseline assessments simultaneously, it is not possible for study staff to know what group participants will be assigned to until after baseline assessments are completed.

### Blinding

Participants and study staff are unaware of group allocation at the time of recruitment and baseline assessments because randomization is performed after participants have completed baseline assessments. However, there is no blinding to group allocation after randomization. Participant blinding is difficult to achieve because intervention group participants are aware of the support they receive, whereas usual care group participants are told to follow their usual routine. This said, participants are blinded to the hypotheses of this trial. Though it is possible that a lack of blinding may lead to exaggerated estimates of intervention effects, a meta-epidemiological study of 146 meta-analyses including 1,346 trials with a wide range of interventions and outcomes found little evidence of bias in trials with objective outcomes when there was a lack of blinding [44]. Study staff and PA counsellors must also be aware of participants’ group allocation after randomization because they are coordinating data collection and intervention implementation. To minimize bias, study staff are not collecting quantitative outcome data; self-report data are collected through a web-based platform and PA is assessed objectively.

### Sample size

A conventional power calculation is inappropriate for this pilot RCT with primary feasibility and acceptability outcomes [45, 46]. Rather, recruitment will remain open until 30–40 young adults (i.e., 15–20 per group) consent and are randomized as per recommendations for pilot trials. This represents 15–20% of the anticipated target sample size for a large, full-scale trial in which 100 participants per group are required to detect a small effect of 0.2 in the primary effectiveness outcome (i.e., PA behavior) with 90% power and two-sided 5% significance [46], and accounting for a 30% dropout rate (i.e., approximating 68 participants per group).

### Procedures

Study staff screen potential participants by phone to ensure they meet eligibility criteria. Following confirmation of eligibility and prior to any data being collected, potential participants are invited to provide informed consent digitally through a web-based form housed on SurveyMonkey. Afterwards, they are enrolled into the pilot RCT by study staff and asked to complete baseline assessments, which consist of wearing an accelerometer for 7 days to assess PA behavior (primary effectiveness outcome) and completing an online questionnaire package consisting of validated patient reported outcome measures (secondary effectiveness outcomes). Once baseline assessments are completed, participants are informed by study staff if they have been randomized to the intervention group or the usual care group; regardless of group allocation, the medical care participants receive during the ACCESS trial is determined by their healthcare team and no concomitant treatments are prohibited. All participants (regardless of adherence to the intervention) are then asked to complete post-intervention assessments, which consist of wearing an accelerometer for 7 days, completing an online questionnaire package, and taking part in an interview (to discuss, in part, acceptability). At follow-up, all participants are again asked to wear an accelerometer for 7 days and complete an online questionnaire package. To prompt completion of post-intervention and follow-up assessments and maximize data collection, emails are sent and/or phone calls are made to all participants.

In addition, feasibility metrics (as described below) are tracked by study staff throughout the trial and notes are taken by PA counsellors. At trial cessation (or when their contract ends), PA counsellors will be invited to take part in an interview by phone or videoconferencing technology (after they provide verbal consent). See Table 1 for schedule of assessments. The CONSORT flow diagram, as presented in Fig 1, will be completed and presented in forthcoming publication of trial results to summarize the process of recruitment and follow-up of participants within the ACCESS trial.

**Table 1:** Schedule of assessments and measures.

Measures (location)	Throughout the trial	Baseline / pre-intervention	Post-intervention	Follow-up	Trial cessation (or when PA counsellor’s contract ends)
		(Week 0)	(Week 13)	(Week 24)	
Personal and medical questionnaire (web-based platform)		X	X*	X*	
Health and wellbeing questionnaire (web-based platform)		X	X	X	
SDT constructs questionnaire (web-based platform)		X	X	X	
BCT questionnaire (web-based platform)		X	X	X	
Accelerometer (field-based)		X	X	X	
Participant interview (phone or videoconference)			X		
PA counsellor ‘exit’ interview (phone or videoconference)					X
Feasibility metrics tracking by study staff	X				
Adverse events tracking by study staff	X				

*Notes*. SDT = self-determination theory; BCT = behavior change technique; PA = physical activity.

*Certain questions for which responses are not expected to change are not included at post-intervention and follow-up assessments.

#### The ACCESS intervention (intervention group)

Participants randomized to the intervention group receive six real-time, one-on-one 60–minute videoconference sessions with the same PA counsellor over a 12-week period. See Table 2 for an overview of the session topics.

**Table 2:** ACCESS intervention session topics and activities/worksheets.

Sessions	Topics	BCT groupings*	Activities/worksheets
1	Introduction and discovery Welcome and introductions Discuss the benefits of PA and risks of inactivity Discuss goal setting for PA and action planning Discuss PA self-monitoring techniques	Shaping knowledge Natural consequences Goals and planning Feedback and monitoring	1.1 Current PA behaviors 1.2 PA guidelines 1.3 SMART goals 1.4 Action plan #1 1.5 Self-monitoring strategies
2	Setting up for success Discuss what is and is not PA Discuss PA barrier identification and management Update action plan	Shaping knowledge Comparison of outcomes Antecedents Goals and planning	2.1 Pros and cons worksheet 2.2 Stages of change model 2.3 Exploring different types of PA 2.4 Barrier identification 2.5 Barrier management 2.6 Action plan #2
3	Getting going Discuss social support for PA Discuss time management Update action plan	Shaping knowledge Social support Natural consequences Goals and planning	3.1 Types of social support 3.2 Identifying sources of support 3.3 Understanding time management 3.4 Action plan #3
4	Adjusting your perspective Discuss how PA is related to mood Discuss environmental restructuring Update action plan	Shaping knowledge Regulation Comparison of outcomes Feedback and monitoring Antecedents Goals and planning	4.1 Progress Review Worksheet 4.2 Increasing enjoyment 4.3 Journaling your feelings 4.4 Environmental restructuring 4.5 Action Plan #4
5	Reinforcing new behaviors Encourage identifying self as a PA role model Discuss framing/reframing PA Update action plan	Shaping knowledge Self-beliefs Goals and planning	5.1 Progress review and problem solving 5.2 Examining the new you 5.3 Reframing self-talk 5.4 Action plan #5 *Materials from previous sessions* 2.2 Stages of change 3.3 Big picture
6	Keep going Review topics covered throughout the program Explore how PA fits into life long-term Revise or create a new action plan for the next step in PA journey Discuss strategies for managing stress Re-iterate that PA journey is not linear	Shaping knowledge Regulation Goals and planning	6.1 Stress management information sheet 6.2 Progress report 6.3 Long-term action plan

*Notes*. PA = physical activity, referring to all movement including during leisure time, for transport, and as part of one’s work. BCT = behavior change technique.

*BCT groupings refer to the hierarchically-clustered 93 techniques presented in the BCT taxonomy (v1) [47].

The ACCESS intervention is multicomponent and includes essential elements derived from previous studies with young adults who have completed cancer treatment [18], as well as from published interventions yielding significant changes in PA [48]. The intervention targets SDT constructs, focusing specifically on: (1) providing autonomy support, structure, and interpersonal involvement; (2) increasing perceptions of autonomy (i.e., perceived control over one’s actions by providing a rationale, structure, and emphasising responsibility), competence (i.e., perceived mastery of tasks and skills by providing support and encouragement, information feedback, and support barrier identification), and relatedness (i.e., perceived belonging and connection to others by encouraging social support seeking); and (3) increasing autonomous motivation (i.e., acting through self-endorsement and volition because the activity holds inherent interest and/or personal value). Accordingly, the intervention includes several content and relational techniques to augment these SDT constructs and facilitate PA behavior change. Techniques were selected based on the theoretical construct they are proposed to target, wherein we drew on published literature [10, 22, 23, 49] to draw out which techniques target specific SDT constructs. BCTs used to guide the content of the intervention and inform the development of activities/information sheets are presented in Table 2. Moreover, PA counsellors use MBCT and motivational interviewing (MI) techniques that align with SDT [22] for the relational component of the intervention to support the delivery of the content; these are presented in Table 3. As content and relational techniques are interrelated, there is overlap between some techniques presented in Tables 2 and 3. By using MI techniques, PA counsellors aim to help participants explore and resolve their ambivalence toward PA behavior change, and thus they tailor the content of the intervention to match participants’ readiness to change, perceived advantages/disadvantages for change, and self-efficacy beliefs [50]. To this end, PA counsellors avoid argumentation and “roll with resistance,” acting as an engaged problem-solving partner [51]. MI principles and techniques used by PA counsellors, which align with SDT [49], include empathic/reflective listening, asking open-ended questions, prompting participants to ask questions, prompting participants to offer solutions, seeking participants’ permission to provide information and advice, shifting focus, supporting change/persistence, and showing unconditional regard.

**Table 3:** Overview of ACCESS intervention components organized by primary SDT construct.

Primary psychological need targeted	Relational techniques	Descriptions
Autonomy	Provide choice (MI/MBCT)	PA counsellor offers choices and options
	Acknowledge participants’ perspectives on behavior (MI/MBCT)	PA counsellor takes time to understand participants’ perspectives on PA and recognize their challenges and efforts. PA counsellor encourages participants to explore and share their perspectives on the causes and perpetuating factors of their current PA behavior
	Provide a meaningful rationale (MBCT)	PA counsellor provides a meaningful rationale for undertaking PA
	Use of non-controlling, informational language (MBCT)	PA counsellor uses informational, non-judgmental language that conveys freedom of choice, collaboration, and possibility when communicating with participants
	Intrinsic goal orientation (MBCT)	PA counsellor encourages identification of intrinsic goals
	Explore life aspirations and values (MBCT)	PA counsellor has participants identify and list important life aspirations, values and/or long-term interests, and has them explore how changes in PA could be linked to them
	Provide structure (MI)	PA counsellor sets parameters within which choice and agency can take place and provides support to initiate action
	Encourage self-experimentation and self-initiation of behavior (MBCT)	PA counsellor prompts participants to experiment and self-initiate PA that could be fun and enjoyable, experienced as a positive challenge, seen as an opportunity for learning or personal expression, and/or associated with skill development
	Emphasize responsibility (MI)	PA counsellor encourages participants to take on responsibility in decision making and/or leadership
	Prompt identification of sources of pressure for behavior change (MBCT)	PA counsellor helps participants identify possible sources of pressures and expectations
	Explore reasons (MI)	PA counsellor explores participants’ reasons for changing their PA behavior
Competence	Offer constructive, clear, and relevant feedback (MBCT)	PA counsellor provides specific relevant, tailored, non-evaluative feedback on participants’ PA goal progress and behavior
	Provide informational feedback (MI)	PA counsellor provides feedback offering information of *how* participants achieved/did not achieve a desired outcome, rather than generic praise/criticism
	Barrier identification (MI)	PA counsellor works with participants to identify barriers to PA behavior change and identify ways of overcoming them
	Assist in setting optimal challenge (MBCT)	PA counsellor assists participants in identifying goals that are realistic, meaningful, challenging, and achievable
	Help develop a clear and concrete plan of action (MBCT)	PA counsellor and participants develop an action plan to work toward a PA behavior goal
	Clarify expectations (MBCT)	PA counsellor has participants discuss their expectations in terms of PA behavior change (i.e., has them identify goal(s)), both its experiential elements (process) and outcomes
	Promote self-monitoring (MBCT)	PA counsellor promotes monitoring of progress, and discusses options for monitoring tools/means and metrics for success
	Address obstacles for change (MBCT)	PA counsellor helps participants identify likely barriers to PA behavior change, and explore how to overcome them
	Explore ways of dealing with pressure (MBCT)	PA counsellor discusses ways to manage and limit effects of pressuring contingencies that would undermine perceptions of competence for PA (e.g., extrinsic rewards, criticism, negative feedback)
Relatedness	Express a personal interest in the individual and take time to develop a rapport (MI)	PA counsellor listens to participants and shows personal involvement
	Prompt identification and seek available social support (MBCT)	PA counsellor prompts identification of sources of support for PA behavior change (if relevant), acknowledges challenges in recruiting adequate support (autonomous vs controlled), and promotes effective ways of seeking positive support
	Acknowledge and respect perspectives and feelings (MBCT)	PA counsellor makes statements of empathy, acknowledges participants’ perspectives, conflicts/ambivalence, distress, and negative affect, and expresses positive feelings when communicating with participants
	Provide support and encouragement (MI)	PA counsellor provides general support and encouragement
	Involvement (MI)	PA counsellor expresses a personal interest in participants and takes time to develop a rapport
	Encourage asking questions (MBCT)	PA counsellor prompts participants to pose questions regarding their PA goal progress and behavior
	Show unconditional regard (MBCT)	PA counsellor expresses positive support regardless of participants’ successes or failures
	Demonstrate/show interest in the person (MBCT)	PA counsellor provides statements of interest and curiosity about participants’ thoughts and perceptions, personal history and background, social context, life events, etc. when communicating with them
	Use empathetic listening (MBCT)	PA counsellor demonstrates attentiveness to participants’ responses (e.g., stays silent to allow participants to complete sentences), and provides reflective and summary statements when appropriate (directed at affect or content) when communicating with them. PA counsellor also asks participants for permission to provide new information, guidance, or advice

*Notes*. As previously mentioned, techniques may support more than one need, and it is implied that techniques used to foster needs satisfaction will also help to foster autonomous motivation [22]. MI = motivational interviewing. MBCT = motivation and behavior change techniques. PA = physical activity.

*Training and ongoing supervision of PA counsellors*. Owing to the nature of the intervention, PA counsellors who are current or past graduate students in kinesiology or a related field are hired. PA counsellors are trained, supervised, and supported by the first author (who holds a PhD in PA and health psychology) and the second author (who holds a masters in Human Kinetics). PA counsellors complete a 2–hour training session (conducted by the first and second authors), are observed performing a mock counselling encounter, and take part in a bracketing interview to examine how their own backgrounds, perceptions, and interests could impact them in their role. Ongoing supervision consists of 30–minute team meetings every 2 weeks focused on updates on participants’ progress, discussion of challenging sessions, deviations or modifications to sessions, and feedback on session notes. During these meetings, PA counsellors are invited to reflect on and discuss their experiences conducting the sessions. Of note, these meetings also serve to monitor data on outcomes, accrual, and adverse events, and oversee participants’ safety as a data monitoring committee is not needed for the ACCESS trial because the intervention is non-invasive with minimal risk of harm.

#### Usual care group

Usual care as a comparison group is necessary as the aim of a future large, full-scale RCT will be to compare the ACCESS intervention with *de facto* usual care (or treatment as usual). Without it, it would not be possible to claim that the ACCESS intervention be the new standard of care. Participants randomized to the usual care group are asked to continue their usual activities of daily living. No restrictions are placed on their PA behavior. Once participants complete follow-up assessments, study staff email them a copy of the intervention materials to review on their own in hopes that it could possibly lead to increases in PA even though they do not have an opportunity to engage with a PA counsellor. Another reason to provide participants randomized to the usual care group with a copy of the intervention materials once they complete follow-up assessments is to promote retention.

### Measures

#### Pilot RCT primary outcomes

*Feasibility [throughout the trial]*. Drawing on the CONSORT 2010 statement for pilot and feasibility trials [42] and CONSORT flow diagram, the following feasibility aspects are tracked by study staff: (1) number of months needed to reach target sample size; (2) number of young adults assessed for eligibility (with referral source) and reasons for exclusion (i.e., not meeting inclusion criteria, declined to participate, other reasons); (3) number of young adults who consented and reasons for refusal; (4) number of participants randomized and reasons for refusal; (5) number of participants allocated to the intervention group and the usual care group; (6) number of intervention sessions attended and proportion of participants who adhered to the intervention (i.e., attended all sessions); (7) number of participants who discontinued the intervention and reasons; (8) number and type of adverse events related to the intervention and/or trial procedures; (9) number of participants lost to follow-up (i.e., who do not complete post-intervention and follow-up assessments) and reasons; (10) number of participants analyzed, number excluded from analysis, and reasons; and (11) percentage of missing data on quantitative measures and number of participants who completed an interview. Study staff conduct a biweekly audit of these metrics (along with adverse events, if any); the first author/principal investigator reviews these during biweekly meetings.

Criteria were set a priori based on relevant literature and the authors’ experiences to determine feasibility of the intervention and trial protocol. The intervention and trial protocol will be considered feasible if: (1) target sample size is reached within 20 months; (2) 2–3 young adults are referred or self-refer per month and are assessed for eligibility; (3) ≥70% of eligible young adults consent and are randomized, with 50% being allocated to the intervention group and 50% to the usual care group, and ≤10% discontinuing the intervention; (4) ≥75% of participants in the intervention group complete all six videoconference sessions for an average of ≥50 minutes; (5) ≤25% of participants are lost to follow-up (i.e., ≥75% complete post-intervention and follow-up assessments) allowing for ≥22 participants (≥11 per group) to be analyzed; (6) <10% missing data on quantitative measures and ≥75% of participants are interviewed; and (7) no adverse events related to the intervention are reported. These data will serve to determine if/what changes/modifications are needed to improve the intervention and/or trial protocol, and will inform the timeline and budget for a large, full-scale RCT.

*Acceptability–participants [post-intervention period]*. All participants are invited to take part in an audio-recorded, semi-structured interview by phone or videoconferencing technology to share their opinions about the trial protocol. Additionally, participants in the intervention group are asked questions related to the intervention (i.e., content, delivery mode, length, duration, PA counsellor), SDT constructs, and BCTs/MBCTs. Their feedback will serve to determine if/what changes/modifications are needed to improve the intervention and/or trial protocol. It will also offer insight into the impact of the various intervention components in achieving the overall outcome of increased PA.

*Acceptability–PA counsellors [at trial cessation or contract termination]*. Each PA counsellor will be invited to take part in an audio-recorded, semi-structured interview by phone or videoconferencing technology to explore their experiences delivering the ACCESS intervention, as well as their thoughts about the content of the intervention, required training, ongoing supervision, using an intervention manual, conducting sessions using videoconferencing technology, maintaining fidelity to the manual, and their motivation and confidence to conduct the sessions. Their feedback will serve to determine if/what changes/modifications are needed to improve the ACCESS intervention and help to highlight factors that may facilitate or hinder the effectiveness of the intervention and/or its implementation in the future.

#### Large, full-scale RCT effectiveness outcomes

*Primary–PA behavior [baseline*, *post-intervention*, *follow-up]*. Objective PA behavior is assessed using an accelerometer (ActiGraph GT9X-BT [also known as ActiGraph GT9X Link]). Total volume of PA (i.e., overall number of minutes spent engaging in light, moderate, and vigorous intensity PA per week) will be considered rather than only moderate-to-vigorous intensity PA (MVPA) for several reasons: (1) studies have highlighted concerns with generic application of intensity cut points as most were developed from young healthy volunteers, whereas young adults who have completed cancer treatment may have ongoing cancer-related side effects that impact their functional capacity; (2) MVPA may not be appropriate or desirable for certain participants with functional/mobility issues that preclude intensive PA; (3) data show that light intensity PA (not just MVPA) confers health benefits across the cancer spectrum [52]; and (4) using total volume of PA will facilitate comparisons across studies as researchers who have summarized accelerometer data into discrete variables (e.g., light intensity PA, MVPA) cite different cut point algorithms. The primary endpoint is total volume of PA at post-intervention (week 13); the secondary endpoint is total volume of PA at follow-up (week 24). Data are managed with ActiLife v6.13.4 software using established wear time criteria [53] and data processing items will be reported (e.g., definition of non-wear time, filters applied during processing, valid day definition and minimum number of valid days, epoch length, vectors used during processing) in forthcoming publication of trial results. Compliance with accelerometer wear will also be reported. Accelerometer based PA measurements taken in free living conditions have been shown to be reliable and valid [54–56]. These data will be used to produce standard deviation estimates to inform the sample size calculation for a large, full-scale RCT.

*Secondary–patient reported outcomes [baseline*, *post-intervention*, *follow-up]*. Patient reported outcomes are assessed using the following questionnaires: RAND 36-Item Short Form Health Survey [57, 58], Positive and Negative Affect Schedule Short Form (PANAS-SF; [59]), Patient Health Questionnaire Depression Scale (PHQ-9; [60]), Patient Health Questionnaire Generalized Anxiety Disorder Scale (GAD-7; [61]), and the Impact of Cancer Instrument–Adolescent and Young Adult Module [62]. These data are collected because they represent secondary outcomes for a large, full-scale RCT, and thus the suitability of the measures and the methods for data collection must be tested.

*Secondary–putative mechanisms of PA change [baseline*, *post-intervention*, *follow-up]*. Testing mechanisms of behavior change is central for a large, full-scale RCT to assess how the intervention works [63, 64]. Thus, SDT constructs are assessed at each timepoint using the following measures to ascertain feasibility of data collection: Psychological Need Satisfaction in Exercise Scale [65, 66] modified to assess basic psychological needs satisfaction in relation to PA, Behavioural Regulation in Exercise Questionnaire [67, 68] modified to assess autonomous motivation for PA, and the Health Care Climate Questionnaire [69] modified to assess autonomy support, structure, and interpersonal involvement in the context of the intervention sessions. The Health Care Climate Questionnaire is only included in the post-intervention questionnaire and is only be administered to participants in the intervention group as the items are irrelevant for participants in the usual care group who do not have a PA counsellor (e.g., “*My PA counsellor conveys confidence in my ability to make changes regarding my PA behavior*”). Also, a questionnaire covering BCTs/MBCTs was developed by the research team and is administered to assess which specific techniques and strategies participants used to change their PA behavior.

#### Additional measures

*Personal and medical factors [baseline unless otherwise specified]*. At baseline, participants self-report a range of personal (age, biological sex, gender identity [using categories currently employed by Statistics Canada in the Census that are based on wording from the Employment Equity Act], race, civil status, work status, education attainment, income) and medical information (height, body mass, cancer type and stage, cancer treatments received, current medication use, comorbid conditions using the Cumulative Illness Rating Scale; [70]). At post-intervention and follow-up assessments, they are asked to self-report potentially changing personal and medical information (i.e., civil status, work status, education attainment, income, comorbid conditions, body mass, current medication use), as well as use of other resources (e.g., personal trainer, psychosocial support). These personal and medical data are collected to describe the sample and assess if such factors impact feasibility outcomes.

*Adverse events [throughout the trial]*. Becoming more physically active is very safe for most young adults after cancer treatment ends and can yield many health benefits [71, 72]. Though the risk of injury is estimated to be very low in this trial, PA counsellors educate participants on the warning signs that may indicate a problem as per Canadian Society for Exercise Physiology guidelines (e.g., chest discomfort, unusual shortness of breath, dizziness or light-headedness, heart rhythm abnormalities) and tell participants to seek immediate medical attention should one of these signs occur. Adverse events (i.e., any unfavourable and unintended sign, symptoms, or disease) definitely, probably, or possibly related to engaging in PA or trial procedures (e.g., wearing an accelerometers) will be tracked. To this end, all adverse events occurring during trial participation are documented by PA counsellors. Each session, PA counsellors record any adverse event(s) participants share (if any) in their notes and report them immediately to the principal investigator via email. At the time of reporting, the intervention is paused for the participant reporting the adverse event until clearance from an appropriate healthcare provider to resume the intervention is obtained. All adverse events reported will be shared with Institutional Review Boards and reported in forthcoming publication of trial results. Additionally, there is a risk that participants experience distress in response to certain questions included in the questionnaire packages and/or posed during the interviews; participants receive a list of free resources to consult in case this occurs during the consent process (e.g., Canadian Cancer Society–Peer support / cancer information specialist: 1-888-939-3333).

*Intervention fidelity [throughout the trial]*. The assessment of fidelity (i.e., the extent to which PA counsellors were able to follow the intervention protocol as intended) is critical when counsellors use an intervention manual so as to ensure the intervention delivery is standardized with limited variation [73]. However, the intervention is delivered by persons (i.e., not automated), and their personal backgrounds, perceptions, and interests may influence their interactions with participants. Therefore, as mentioned above, each PA counsellor participates in a bracketing interview with the second author. The goal of the bracketing interview is to help them explore and address any biases that may inadvertently influence how they deliver the intervention content and interact with participants. In addition, following each session with participants, PA counsellors take detailed notes regarding required deviations, implementation facilitators and challenges (including strategies used to overcome challenges), participants’ responses, external influences on the intervention, and any factors they believe may have influenced their ability to follow the intervention protocol (i.e., intervention fidelity). These notes are reviewed during the biweekly meetings to facilitate fidelity monitoring, and will be synthesized to provide a broad assessment of the intervention implementation process.

### Data management and analysis

#### Main analyses

Quality checks and content validation will be utilized to ensure high quality data (i.e., identify sources of inconsistency, checking for errors). Quantitative and qualitative data analyses will be conducted. SPSS and SAS will be used for quantitative data management and analysis; NVivo will be used for qualitative data management and analysis. Descriptive statistics will be computed to describe the sample based on baseline personal and medical factors, and report on feasibility and acceptability outcomes described above. Specifically, categorical variables will be described using proportions and frequencies, and continuous variables with means and standard deviations (or with medians and interquartile ranges for data not normally distributed). Content analysis [74] of the transcribed interviews will be conducted to identify themes and see if/what changes to the intervention and/or trial protocol are warranted.

#### Additional analyses

This pilot RCT is not powered to determine the effectiveness of the ACCESS intervention, which is why the main analyses will focus on feasibility and acceptability outcomes. Nonetheless, effectiveness outcomes will be analyzed descriptively to inform the design of the future large, full-scale RCT. The primary effectiveness outcome (i.e., objectively measured PA behavior assessed at baseline, post-intervention, and follow-up) will be analyzed by fitting a linear regression model accounting for the correlation in repeated measures on the same participant over time explicitly modeling a covariance matrix that best fits the data based on the likelihood ratio tests and information criteria. The model will include the stratification factor (i.e., biological sex), PA counsellor ID, and characteristics commonly associated with attrition (e.g., length of chemotherapy) as covariates under the assumption of missing at random. The model allows all available observations on each participant to be used without having to use an imputation procedure. The model will be estimated by means of Restricted Maximum Likelihood (REML) and used to produce estimates of usual care group means and event rates, and variance and correlation estimates to inform the sample size calculation for a large, full-scale RCT. Degrees of freedom will be computed using the Kenward-Roger approach [75].

Additionally, the amount and potential richness of data collected facilitates supplementary exploratory analyses. Therefore, after the main analyses are conducted to address the pilot trial objectives, it is envisaged that exploratory analyses may be undertaken to address subsequent research objectives that will make unique theoretical and empirical contributions (e.g., changes in secondary outcomes).

### Dissemination plans

Trial results will be shared with academic and non-academic audiences via presentations at scientific meetings and community meetings, as well as through publication of manuscripts in peer-reviewed journals. Contributors who make substantive intellectual contributions to presentations and manuscripts will be given credit as authors, provided they meet criteria for authorship outlined by target journals (or outlined herein, if none provided: http://www.icmje.org/recommendations/browse/roles-and-responsibilities/defining-the-role-of-authors-and-contributors.html#two’). Additionally, results will be submitted to clinicaltrials.gov no later than 1 year after trial completion. Finally, the principal investigator posts trial information and updates on her website (https://www.pahealthpromolab.com), and will post a summary of results on her website; participants are informed of the team’s intent to do this by study staff.

### Trial status

Whilst the advent of Covid-19 presented significant challenges and delayed the start of recruitment, it also provided a valuable opportunity to consider alternate methods of collecting data (e.g., sole reliance on methods to collect data remotely; online questionnaires, mailing accelerometers, interviews by phone or videoconferencing technology). Recruitment started in February 2021. As of May 5, 2022, 63 young adults had been screened and 39 had been enrolled, of whom 37 had been randomized. The approximate date of recruitment completion is October 1, 2022 (or possibly later). The protocol reported here is version 7 and was last updated on July 19, 2021.

## Discussion

PA is a promising non-pharmacological, non-invasive, and cost-effective intervention that confers several health benefits for young adults who have completed cancer treatment [71, 72]. It has been further shown that PA can help to reduce the risk of morbidity and early mortality in this population [4, 5]. Yet, PA levels are low after cancer treatment ends [6–8, 76]. Increasing the uptake and maintenance of PA throughout survivorship is therefore a public health priority.

There are many barriers to engaging in PA, including a lack of support for PA [32, 77]. Most studies that have developed, implemented, and evaluated a PA intervention for young adults who have completed cancer treatment are based on formal PA programs (e.g., supervised exercise; [27]). Whilst necessary to assess the effects of PA, these interventions generally require that young adults set aside time to attend sessions in person at a designated location. As well, these interventions seldom teach young adults how to integrate PA into their daily lives based on their personal goals and interests. By contrast, providing behavioral support may be effective at empowering young adults to develop personal goals and learn how to integrate PA into their daily lives. This approach addresses young adults’ mindset about PA, as well as their motivation for engaging in PA with the goal of supporting the uptake and maintenance of PA [78–80]. Such an approach is compatible with SDT and has been shown to support the uptake and maintenance of PA in experimental studies [9, 18, 27, 81]. Therefore, novel, SDT-driven behavior change interventions that use empirically based BCTs/MBCTs and address participation barriers by offering real-time sessions via videoconferencing technology, like the ACCESS intervention presented herein, could help to promote PA in young adults who have completed cancer treatment. However, research is needed to confirm this hypothesis.

Before the effectiveness of the ACCESS intervention can be investigated in a large, full-scale RCT, it is first necessary to ascertain that it can be delivered and carried out as intended. To this end, a pilot RCT is ongoing to: (1) assess intervention and trial protocol feasibility and acceptability; and (2) generate data on PA behavior. The results of this pilot RCT will determine uncertain parameters needed to design and implement a large, full-scale effectiveness RCT, including the variance in PA behavior change needed for sample size calculation, ability to deliver the intervention as intended, participants’ and PA counsellors’ acceptability, adherence and compliance, recruitment, enrollment, retention, loss to follow-up, intervention discontinuation rates, and data completeness.

This manuscript describes the ACCESS trial and all relevant elements of the intervention. The ACCESS intervention has the potential to serve as a model to optimize the delivery of behavior change interventions to promote PA. Strengths include its pragmatic design–namely broad enrollment criteria, use of various local and national recruitment strategies, use of distance-based means to collect data to encourage participation of young adults near and far from urban centres, and use of technology to deliver the intervention to address participation barriers (e.g., lack of time, unwillingness to travel for sessions). These key features will allow for future implementation of the trial in real world systems that do not rely on a single centre or geographic location for recruitment. In addition, through a combination of quantitative and qualitative methods, it will be possible to evaluate the intervention and trial protocol in greater depth than if only looking at feasibility as assessed by quantitative metrics.

## Conclusion

There are over 18,000 young adults (aged 18–39 years) diagnosed with cancer per year in Canada [81] and 1.2 million globally [82]. Cancer care, whether delivered in hospitals or community centres, is typically located in urban areas and behavior support for PA is not always prioritized. Given the ubiquity of the Internet, the ACCESS intervention could be implemented across and beyond Canada to support efforts to promote PA in this growing, mostly insufficiently active segment of the population. As a first step, it is important to consider whether the ACCESS intervention, and methods proposed to evaluate its effects, are feasible and acceptable. Future research may then look at its effectiveness and costs to support the identification of a quality, cost-effective intervention to promote PA.

## Supporting information

S1 File SPIRIT 2013 checklist: Recommended items to address in a clinical trial protocol and related documents*. (DOC)

S2 File (PDF)

## Abbreviations

ACCESS: The physicAl aCtivity Counselling for young adult cancEr SurvivorS trial
BCTs: Behavior change techniques
CONSORT: Consolidated Standards of Reporting Trials
MBCTs: Motivation and behavior change techniques
MI: Motivational interviewing
MVPA: Moderate-to-vigorous intensity physical activity
PA: Physical activity
RCT: Randomized controlled trial
SDT: Self-determination theory
TOH: The Ottawa Hospital
