# Supplementary material for: The physicAl aCtivity Counselling for young adult cancEr SurvivorS (ACCESS) trial: A protocol for a parallel, two-arm pilot randomized controlled trial
Source: PLoS One. 2022 Dec 30;17(12):e0273045. doi: 10.1371/journal.pone.0273045 (PMC9803096; doi:10.1371/journal.pone.0273045)
Supplement: S2 File — (PDF) [file pone.0273045.s002.pdf]

Title: Physical activity counselling for young adult cancer survivors.

Investigators:
Principal Investigator
Jennifer Brunet, PhD, University of Ottawa

Study Summary/Abstract
Survival rates for cancer are increasing, resulting in more young adult cancer survivors (YACS) living with lingering side effects. Physical activity (PA) has been shown to improve the quality and length of life in YACS; yet, few are active enough and currently little is being done to promote PA in this population. We developed a novel behaviour support intervention to promote PA in YACS. Before conducting a large-scale randomized controlled trial (RCT) aiming to test the effects of a behavioural support intervention in comparison to usual care (UC) in YACS, a pilot-RCT is necessary to determine whether the intervention delivered via videoconferencing is both feasible for and acceptable to YACS. In this two-arm pilot RCT, we aim to: (1) assess trial and intervention feasibility, (2) assess trial and intervention acceptability, and (3) generate data on PA behaviour. Thirty to forty YACS will be randomized to receive either a 12-week behaviour support intervention delivered via videoconferencing (group A) or will be allocated to usual care (no intervention; group B). The main feasibility outcomes will be recruitment, adherence, retention and missing data rates which will be tracked by study staff. Acceptability will be assessed through interviews exploring participants' experiences, thoughts, and perspectives of the trial (Groups A and B), as well as their views of the intervention and its mode of delivery (i.e., videoconferencing; group A only). PA behaviour will be measured using accelerometers, provided at baseline, tracking PA throughout and after the intervention. Feasibility and acceptability data will help determine if/what changes/modifications are needed to improve the trial and/or intervention, and will inform the timeline and budget for a definitive RCT. The PA behaviour data collected in the trial will inform sample size calculation for a future RCT that aims to test the effects of our intervention in YACS.

Research Purpose and Objectives: This study, in which YACS are randomized to receive either a behaviour support intervention delivered using real-time videoconferencing or usual care, aims to:

1. Assess trial and intervention *feasibility*;
2. Assess trial and intervention *acceptability*;
3. Generate *estimates of variance* in objective PA behaviour to inform sample size calculations for a definitive RCT.

Introduction/Background:

In Canada, over 10,000 people aged 18-39 years are diagnosed with cancer annually.[1] YACS report more physical disability, psychosocial dysfunction, disrupted educational and vocational attainment, and poorer quality of life than middle-age and older adults.[2-10] Despite the need to develop interventions to reduce the cancer burden in YACS,[11] very little research has focused on YACS. The work that has been conducted has been observational in nature and of low quality (e.g., underpowered, high bias). Accordingly, research has

not developed, implemented, and evaluated intervention or examined the best way to deliver interventions to this population. Also, most researchers have not focused on early survivorship – a transitional period marked by treatment cessation (i.e., 'teachable moment') wherein individuals are more prone to changing their behaviors and habits. This is problematic as YACS have difficulties regaining prior levels of function,[11-13] and wish to make lifestyle changes to prevent mortality and morbidity during this time.[14]

PA promotes survival, reduces the risk of disability, and improves quality of life in cancer survivors which in turn reduces the burden of cancer.[15-18] PA guidelines have been issued for cancer survivors;[19-21] yet, most YACS do not meet PA guidelines and PA levels are often too low to accrue health benefits.[22] Efforts to promote PA in YACS are severely lacking.[12] Since YACS are a distinct group with unique preferences, needs, motives, and goals,[23] we conducted a scoping review to identify PA determinants in YACS,[12] followed by a qualitative study to understand their preferences, motives, and needs for PA interventions to inform the development of a behaviour change intervention.[24] From this work, we concluded that psychological (e.g., competence, autonomy, relatedness, motivation) and social (e.g., support) factors impact PA behaviour in YACS. Our findings, coupled with other empirical data,[13,25-29] highlight the need to target these factors when developing, implementing, and evaluating PA behaviour change interventions for YACS.

Using theory to develop interventions is vital to guide the choice of intervention components and evaluative measures.[30] SDT is widely used for this purpose because of its focus on psychological and social factors.[31-34] Within SDT, the satisfaction of three basic psychological needs for competence, autonomy, and relatedness are predicted to influence behavioural outcomes directly and indirectly (by promoting autonomous motivation and engagement).[31] It is also argued that those delivering interventions can utilize autonomy support, structure, and interpersonal involvement to satisfy participants' psychological needs and foster autonomous motivation.[31,35] Autonomy support occurs when participants are given authentic PA choices, pressure is minimized, and participants' perspectives are acknowledged.[35,36] Structure is perceived when clear expectations are communicated and participants have access to personalized information and meaningful feedback.[35,36] Interpersonal involvement is fostered when there is trust and a meaningful relationship between the people delivering the intervention (e.g., PA counsellors) and participants.[35,36] Systematic reviews and empirical studies in middle-age and older cancer survivors provide support for using SDT to develop PA behaviour change interventions.[32,37-39] When extended to YACS, findings suggest that interventionists should consider cultivating autonomy support, providing structure, and establishing strong interpersonal relationships with YACS to increase PA behaviour via psychological need satisfaction and increased autonomous motivation. Gillison and Standage conducted a meta-analysis to identify the full range of behaviour change techniques (BCTs) used to promote need satisfaction and autonomous motivation, including BCTs based on SDT or the wider field of behaviour change.[40] The use of such theoretically-informed techniques was found to bring about changes in the psychological precursors of behaviour change of small (i.e., relatedness, motivation), medium (i.e., competence), and large (i.e., autonomy support and satisfaction) effect sizes.

Studies with cancer survivors confirm that PA interventions using BCTs are effective with this population,[41,42] and that those reporting the systematic use of BCTs are more effective than those that do not.[42,43] While cancer-specific websites recognize this and now share information on BCTs,[44] there is little evidence of which BCTs are the most effective in this

setting. This study will address a gap in the literature by exploring the feasibility and acceptability of a theoretically-informed intervention, detailing the specific BCTs predicted to drive the process of change to promote PA in YACS (see Appendix A for Conceptual Model).

Real-time videoconferencing (i.e., the provision of professional, tailored counselling services through the Internet) has gained popularity as a potential mode of intervention delivery and may be ideal for tech-adept YACS who report heavy reliance on the Internet for access to health information and who desire interactive and trustworthy content.[25,27,28,45-49] This intervention delivery mode can also address barriers related to participation (e.g., travel restrictions, scheduling difficulties) and can reduce healthcare provider burden by shifting responsibility of PA promotion to trained PA counsellors.[50,51] Since videoconferencing is an effective way for YACS to learn how to manage symptoms (e.g., pain, anxiety),[52] and behaviour support delivered via videoconferencing promotes health behaviours such as PA in clinical and non-clinical populations,[53-58] the World Health Organization now advocates for enhanced technology use in healthcare settings.[59] Yet, no such behaviour support interventions have been tested in YACS. Thus, the feasibility, acceptability, and effects of a theoretically-informed PA behavioural support intervention utilizing BCTs and delivered via videoconferencing remain unknown.

Study Design: This is a parallel two-arm, single-centre pilot-RCT. The two arms are:
1. Group A: behavioural support intervention delivered via videoconferencing;
2. Group B: usual-care (no intervention).

Outcomes will be assessed at:
1. Pre-intervention (Baseline; Week 0)
2. Post-intervention (Week 12)
3. 3-month follow-up (3 months post-intervention; Week 24)

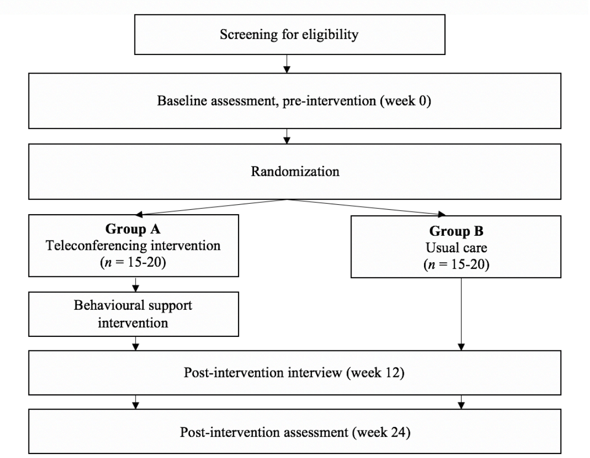

Figure 1. Flow of study

Participants:

Recruitment: Following research ethics board approval at the Ottawa Hospital (TOH), YACS will be recruited from TOH over a 24-month period via physician referral, posters, social media, and word of mouth.

1. **Recruitment strategy 1**: Physician referral
   Dr. Amirrtha Srikanthan (medical oncologist) will oversee recruitment at TOH. They will ask their colleagues to speak to any potentially eligible patients about the study to determine whether they would be interested in learning more by having study staff contact them. Study staff will also present the study at rounds and other forums attended by relevant medical oncologists (once COVID-19 related restrictions have been lifted) to raise the profile of the study and to facilitate recruitment.
   Healthcare providers (i.e., oncologists) will be advised to only refer patients whom they medically clear to participate in the intervention. Healthcare providers will first identify and introduce the study to patients whom they medically clear to participate in the intervention and then ask for the patients permission to be contacted by study staff. The contact information of those patients who have verbally consented to being contacted will be sent by the healthcare provider to the study staff via email (i.e., by sending the patient's name and phone number to study staff in the body of an email). No identifying information will be contained in the email subject; rather patients' names and contact information will be provided in the body of the email only. Patients' names and contact information will be transferred to the secure server at the university and deleted from the email account immediately upon receipt. The study staff will then contact the patient and review their eligibility and obtain informed consent for participation in the study (see Informed Consent section for more details) prior to performing any study-related activities. Healthcare provider referral to the trial will assume medical approval to participate in the intervention (i.e., healthcare providers will only refer those patients whom they medically clear to participate in the intervention).

2. **Recruitment Strategy 2:** Posters, social media, and word of mouth (see Appendix B for recruitment poster).
   Participants will also be recruited via: (1) posters placed in waiting rooms at TOH and the Irving Greenburg Family Cancer Centre, and at established Ottawa cancer survivorship centres (e.g., The Ottawa Regional Cancer Foundation, Ottawa Integrative Cancer Centre); (2) advertisements posted on the principal investigator's research lab website; (3) advertisements placed on YACS organizations websites (e.g., Young Adult Cancer Canada and Localife Ottawa); and (4) word of mouth.

3. **Recruitment Strategy 3:** Recruitment letters sent via mail (see Appendix X for invitation letter).
   Participants will also be recruited via invitation letters mailed to their home address. Letters will be sent to patients who meet inclusion criteria (currently between the ages of 18-39 years; received a first diagnosis of invasive cancer between the ages of 18-39 years; completed primary treatment for cancer <5 years) and who have previously provided permission to be contacted for research purposes via the institutional "permission to contact" process. These letters will describe the goal of the study in lay-terms and what would be involved in participating. Interested participants will have the

option of calling or emailing the research coordinator for more information or to enroll. Additionally, behaviour change research trials typically enroll more participants born as female than participants born as male. Therefore, to encourage more male participants, targeted recruitment letters may be sent to male-born patients only who meet inclusion criteria. These letters will describe the goal of the study in lay-terms, what would be involved in participating, and will specify that there is a need for young adults who were born male to participate in the study specifically to ensure that they are represented in our research. Interested participants will have the option of calling or emailing the research coordinator for more information or to enroll.

Inclusion criteria: All participants will need to meet the following inclusion criteria to participate in the study:

1. Currently between the ages of 18-39 years (per consensus statements and models of care in Ontario);
2. Received a first diagnosis of invasive cancer between the ages of 18-39 years;
3. Completed primary treatment for cancer <5 years;
4. Able to provide informed consent in English or French;
5. Have access to videoconferencing technology (e.g., Skype, Google Hangouts, Facetime)

Exclusion criteria: The following patients will not be eligible to participate in the study:

1. Have evidence of current cancer (recurrent or secondary cancer or relapse);
2. Have physical impairments precluding participation in PA;
3. Currently meeting Canadian Society for Exercise Physiology aerobic PA guidelines for cancer survivors in the month prior to enrollment (i.e., 150 minutes/week of moderate-to-vigorous intensity aerobic PA) assessed over the phone via a self-report screening question;
4. Are non-ambulatory.

Sample size: Calculations were not computed as this is a pilot-RCT with primary feasibility and acceptability outcomes.[65,66] Rather, recruitment will remain open for a 24-month period, with the goal of recruiting 15-20 YACS per group as per recommendations for pilot trials where a definitive trial will be designed with 90% power to detect small effects and two-sided 5% significance, and accounting for a 20-30% dropout rate.[67]

Informed consent: A member of the research team will perform an initial screening of patients by phone to ensure they meet all the inclusion criteria (see Appendix C for recruitment script). Following confirmation of eligibility, patients will be informed that they will receive an email within a 24-48 hours containing a copy of the informed consent form (see Appendix D for English Informed Consent Form). They will be asked to read the consent form, and to sign and send it back to study staff via email. If this option is not available to participants (e.g., they cannot send the signed document back by email), they will be sent a secure link where they will be asked to review the consent form and afterwards provide digital consent (i.e., click 'yes' at the end of the form). Once informed consent (written or digital) has been obtained, participants will be sent a secure link to complete the online survey.

Procedures:

After obtaining informed consent, each participant will complete an online baseline assessment (Week 0) and then will be randomized to one of two groups. Participants in group A will receive the intervention for a 12-week period, whist those in group B will receive usual care. Immediately after this 12-week period and again 12 weeks later (i.e., 24 weeks following intervention initiation), all participants will complete post-intervention and follow-up assessments, respectively. Of note, all assessments (with the exception of the interview) will take place online using Survey Monkey. For the interview, participants will be able to choose whether they want to have the interview over the phone or via videoconferencing.

Randomization**:** Participants will be stratified by sex, and then allocated in a 1:1 ratio by a Clinical Trial Randomization Tool offered by the National Cancer Institute. The randomization schedule will be developed by a member of Dr. Brunet's laboratory who is not involved in this trial.

Behavioural Support Intervention: Group A

Participants in groups A (*n* =15-20) will receive 6 real-time, 1-on-1 60-minute sessions led by a PA counsellor over a 12-week period (see Appendix E for intervention overview and content). Sessions will be delivered via videoconferencing - a dosage commensurate with other interventions that have yielded significant changes in PA behaviour.[63,64] Participants will remain with the same counsellor throughout the intervention to allow for continuity.

Session Aims**:** The aims of the sessions will be to provide YACS with support as well as the knowledge, skills, and confidence to identify and overcome challenges to engaging in PA in order to increase PA behaviour and to enhance patient-reported outcomes. Participants will set their own targets which will aim to progress toward the ACSM aerobic PA guidelines of 150 minutes/week of moderate-to-vigorous intensity PA and 2 days/week of strength training (see Appendix F for PA guidelines handout).

Content: The intervention will target SDT constructs, focusing specifically on: (1) *providing autonomy support*, *structure,* and *interpersonal involvement;* (2) *increasing perceptions of autonomy* (i.e., make participants feel they have control over their lives and behaviour), *competence* (i.e., build participants' knowledge and skills and experience mastery in efforts to build their PA competence), and *relatedness* (i.e., help participants to feel a sense of belongingness with others such as family members, friends, co-workers, and other exercisers) and; (3) *increasing autonomous motivation* (i.e., motivation coming from internal sources and also from intrinsic sources). The intervention will also integrate evidence-based BCTs in order to foster PA motivation and behaviour change.[40] The focus of the BCTs will be: *increasing PA knowledge*, *raising awareness of cancer risks*, *teaching PA benefits*, *increasing PA opportunities*, *offering alternatives*, *identifying and enlisting social support*, *setting intrinsic goals that are measurable, achievable, relevant, and time-related, identifying barriers to PA and formulating plans to remove and overcome barriers*, *teaching self-monitoring and problem-solving*, *providing feedback*, *building confidence and skills*, *rewarding reaching PA goals*, and *changing negative thinking.*

PA counsellors with a BA in Human Kinetics or related field will be hired. PA counselling training and fitness/coaching experience will be considered assets. Given their pivotal role, they will: (1) complete a free online motivational interviewing training module, and; (2) receive an

online training course created by Brunet (Principal Investigator), Fiona Gillison (Co-Investigator), Amirrtha Srikanthan (Co-Investigator), and Amanda Wurz (Co-Investigator).

Usual Care: Group B
Participants randomly assigned to UC group (*n* = 15-20) will be advised to continue with their regular activities of daily living. Participants will not be prevented from participating in PA as it is unethical to prevent PA and is current practice at TOH to provide PA guidelines. Post-trial (week 24), study staff will mail participants in the UC group the same intervention materials that group A received.

Data Collection:
Timing. Outcomes will be assessed at 3 time points:
1. Pre-intervention (week 0)
2. Post-intervention (week 12)
3. 3-month follow-up (12-weeks post-intervention; week 24)

Study participants in both groups will take part in the questionnaire assessments at each timepoint (Table 1), and will all complete an interview about the trial (Groups A and B) and intervention (Group A) acceptability at week 12 (post-intervention).

<u>Study Assessments:</u>
Primary outcomes: trial and intervention *feasibility* and *acceptability*. The primary efficacy outcome for a definitive RCT is objective *PA behaviour* and the secondary efficacy outcomes are *patient-reported outcomes* and *putative mechanisms of change* (i.e., knowledge, skills, perceived need support, psychological need satisfaction, and motivation). Outcomes will be assessed at baseline (week 0), post-intervention (week 12), and 3-month follow-up (week 24).

Feasibility
Our main feasibility outcomes will be (1) *recruitment rates* (defined as the number of eligible participants who enroll in the trial), and (2) *adherence rates* (defined as the number of intervention sessions attended out of 6); (3) *retention rate* (defined as the number of participants completing all three assessments), and (4) *data completeness* (defined as the percentage of missing data on quantitative measures and the number of participants completing interviews; see Appendix G for trial schematic). As suggested,[65,69] we set criteria a priori for targets on each outcome: (1) >70% enrollment of eligible YACS; (2) >90% of participants complete the 6 videoconferencing sessions; (3) all assessments completed by ≥75% of participants based on past studies with YACS,[70-73] and; (4) <10% missing data.
Acceptability – study participants – qualitative. Semi-structured interviews will be completed by all participants in both groups. Acceptability of the trial will be assessed in group A and B. Because group B will not receive the intervention, acceptability of the intervention and delivery mode will be assessed only in group A. Participants will be able to choose how they want to complete the interview (i.e., over the phone or via videoconferencing). Interviews will include both closed (i.e., quantitative) and open-ended (i.e., qualitative) questions about participants' experiences, thoughts, and perspectives of the trial (i.e., study protocol), as well as the intervention, its delivery mode, and the PA counsellor. Interview questions will also cover SDT constructs and BCTs (see Appendix Ha for interview guide for Group A, and Appendix Hb for interview guide for Group B).

Acceptability – PA counsellors – qualitative. Each PA counsellor hired for the study and who has delivered the intervention at least once will be invited to take part in an audio-recorded semi-structured interview, either over the phone or via videoconferencing, at the completion of the trial (for those who continue with the study for the entire period) or at the end of their contract (for temporary hires). They will be asked their thoughts about the content of the intervention, training and ongoing supervision, using an intervention manual, conducting sessions using videoconferencing, maintaining fidelity to the manual, and their motivation and confidence to conduct the session. Their feedback will serve to determine if/what changes/modifications are needed to improve the intervention and help to highlight factors that facilitate or hinder the effectiveness of the intervention. Interviews will be conducted by a research assistant who is also a PhD student in human kinetics with training in PA counselling. Interviews will be guided by an interview guide (Appendix I) and will last approximately 30 minutes.

Quantitative measures. All of the quantitative questionnaires and assessments have been used and/or validated previously with cancer survivors. The study measures will be collected at all three time points listed above and will be collected using online data collection tools, unless otherwise stated.

PA behaviour. The primary efficacy outcome for a definitive RCT is objective PA behaviour (i.e., minutes spent engaging in light, moderate, and vigorous PA) assessed using accelerometers (see Appendix I for accelerometer instructions) which provide valid and reliable data on PA behaviour in cancer survivors.[74] The ActiGraph GT3X+ (Ft. Pensacola, FL) will be used as it is a gold standard PA monitor.[75-78]

Patient-reported outcomes [week 0, 12, and 24]. Secondary outcomes for a definitive RCT include patient-reported outcomes. These will be assessed online using these questionnaires (see Appendix J): RAND 36-Item Short Form Health Survey;[79-80] Positive and Negative Affect Schedule (PANAS-SF);[81] Patient Health Questionnaire Depression Scale (PHQ-9);[82] 7-item Patient Health Questionnaire Generalized Anxiety Disorder Scale (GAD-7);[83] and the Impact of Cancer Instrument – Adolescent and Young Adult Module.[84] The selected questionnaires are reliable, valid, have good responsiveness to change in cancer survivors of all ages, and have been used in past national cohort studies.[85-95]

Putative mechanisms of change [week 0, 12, and 24]. Testing mechanisms of change is central in a definitive RCT to assess how the intervention works.[96-99] These measures covering SDT constructs will thus be included as secondary outcomes in the online survey (see Appendix K): Behavioural Regulation in Exercise Questionnaire-3 to assess autonomous motivation for PA;[100,101] Psychological Need Satisfaction in Exercise Scale modified to the PA context to assess basic psychological need satisfaction in relation to PA;[102,103] and the Health Care Climate Questionnaire modified to assess autonomy support, structure, and interpersonal involvement in the context of the intervention sessions.[104] The Health Care Climate Questionnaire will only be administered at the end of the intervention (week 12) and will not be administered to Group B as the items are irrelevant for the UC group participants who do not have a PA counsellor (e.g., "*My PA counsellor conveys confidence in my ability to make changes regarding my PA behaviour*"). Also, as the intervention uses evidence-based BCTs,[40-44] questionnaires covering BCTs will be included to assess which specific techniques and strategies participants used to modify their PA behaviour (see Appendix L).[105-107]

Additional outcomes [weeks 0, 12, and 24]. Data on participants' socio-demographic background, medical history, and healthcare resource use will be collected to determine if we have non-proportional representation and require strategies to address this in a definitive RCT.

For *demographic* and *other resource use*, participants will self-report age, gender (socially constructed), ethnicity, civil status, work/education status, income, comorbidities (using the Cumulative Illness Rating Scale),[108] and use of other resources (e.g., personal trainer, psychosocial support). For *medical*, participants will self-report sex (biological), height, weight, cancer type and stage as well as the type and protocol of treatments received for their cancer (i.e., surgery, chemotherapy, radiation, immunotherapy, hormonal), and list of current medication(s). See Appendix M.

Table 1. Assessment schedule for participants in the study.

**ASSESSMENTS**	**ASSESSMENT VISIT 1:** (Pre-intervention)	**ASSESSMENT VISIT 2:** (Post-intervention period)	**ASSESSMENT VISIT 3:** (12-weeks post-intervention period)
**Socio-demographic/ medical information**			
Questionnaire	X	X	X
**Health and Wellbeing**			
Questionnaire	X	X	X
**Self-Determination Theory Constructs**			
Questionnaire (both groups; exception – Health Care Climate	X	X	X

Questionnaire will only be administered to group A at 12 weeks)			
**Behavioural Change Techniques**			
Questionnaire (group A only)	X	X	X
**Total time required**			
	30-45 minutes	30-45 minutes	30-45 minutes
**Assessment location**			
	Online (Survey Monkey)	Online (Survey Monkey)	Online (Survey Monkey)
**Trial and intervention acceptability**			
Interview (both groups; exception – intervention acceptability and delivery mode questions will only be asked to group A)		X	
**Total time required**			
		Approximately 60 minutes for group A and 30 minutes for group B	
**Assessment location**			
		Over the phone or via videoconferencing	

Data Analysis

For feasibility outcomes, data will be summarized using descriptive statistics (i.e., frequencies, percentages). For acceptability outcomes, medians with interquartile ranges will be computed for participants' responses to close-ended questions that range from 1-10 and content analysis of participants' responses to open-ended questions, as well as PA counsellors' responses, during the interviews will be conducted to identify themes and see if/what changes to the trial and/or intervention are warranted.[109] For PA behaviour, responses at baseline (week 0), post-intervention (week 12) and 3-month follow-up (week 24) will be analyzed using a constrained repeated measures ANOVA with fixed terms for time and group by time interaction.[110] The model will be used to produce estimates of variance to inform the sample size calculation for a definitive RCT. The correlation in repeated measures on the same participant over time will be accounted for by explicitly modeling the covariance matrix, with the best-fitting covariance structure decided using likelihood ratio tests and information criteria.[111] Estimated correlations

will also be used to inform the sample size calculation for a definitive RCT. Counsellor effects will be accounted for in the analytical models using random effects. While the analysis will be underpowered to evaluate efficacy, preliminary estimates of least square mean differences between groups post-intervention and at 3-month follow-up will be obtained with 75% and 85% confidence intervals (as 95% confidence intervals are not recommended for these purposes).[112] SPSS and SAS will be used for the quantitative analyses; NVivo will be used for the qualitative analyses.

Study Risks:

There is some risk that participants may experience psychological or emotional discomfort while answering the self-reported questionnaires and discussing personal information and/or previous happenings. Should participants experience psychological or emotional discomfort while recalling personal information and/or previous happenings, they will be directed to contact the Canadian Cancer Society – Peer Support at 1-800-263-6750. As well, participants will be directed to call the Canadian Mental Health Association Crisis Line at 1-800-667-8407, which provides immediate support on a 24/7 basis.

For the intervention there is minimal risk of injury that exists when participating in any PA. Indeed, current guidelines recommend that person's diagnosed with cancer engage in regular PA as it is safe and can help to improve health. Nevertheless, as part of the intervention, PA counsellors educate participants on the warning signs that may indicate a problem (e.g., chest discomfort, unusual shortness of breath, dizziness or light-headedness, heart rhythm abnormalities) and tell participants to seek immediate medical attention should one of these signs occur. All adverse events (i.e., any unfavourable and unintended sign or symptoms) *definitely*, *probably*, or *possibly* related to the intervention or study procedures will be tracked by study staff. If any adverse events are reported, at the time of reported, the intervention is paused for the participant until clearance from an appropriate healthcare provider to resume the intervention is obtained. Additionally, following the guidelines for clinical practice set by the Canadian Society for Exercise Physiology (CSEP) (CSEP, 2011), any adverse events will be immediately reported to the institutional research ethics boards. All adverse events reported to study staff will also be published.

Study Benefits:

All participants enrolled in this study may experience improvements in their PA levels, as well as in their physical and psychological health. Further, they will have the opportunity to learn strategies and to develop knowledge, skills, and confidence to engage in PA which could lead to long-term changes in PA behaviour. There is a chance that participants will not experience any benefits from participating; however, participants in this study will help make a significant contribution to the current PA, supportive care, and oncology literatures in YACS.

References

1. Canadian Cancer Statistics Advisory Committee. *Canadian Cancer Statistics 2016.* Toronto, ON: Canadian Cancer Society;2016.
2. Barr RD, Ferrari A, Ries L, Whelan J, Bleyer WA. Cancer in adolescents and young adults: a narrative review of the current status and a view of the future. *JAMA Pediatr.* 2016;170(5):495-501.
3. Hallquist Viale P. Late effects: focus on adolescent and young adult cancer survivors. J Adv Pract Oncol. 2016;7(1):15-16.
4. John TD, Sender LS, Bota DA. Cognitive impairment in survivors of adolescent and early young adult onset non-CNS cancers: does chemotherapy play a role? *J Adolesc Young Adult Oncol.* 2016;5(3):226-231.
5. Kourie HR, Klastersky JA. Physical long-term side-effects in young adult cancer survivors: germ cell tumors model. *Curr Opin Oncol.* 2017;29(4):229-234.
6. Kwak M, Zebrack BJ, Meeske KA, et al. Trajectories of psychological distress in adolescent and young adult patients with cancer: a 1-year longitudinal study. *J Clin Oncol.* 2013;31(17):2160-2166.
7. Miller KD, Siegel RL, Lin CC, et al. Cancer treatment and survivorship statistics, 2016. *CA Cancer J Clin.* 2016;66(4):271-289.
8. Quinn GP, Goncalves V, Sehovic I, Bowman ML, Reed DR. Quality of life in adolescent and young adult cancer patients: a systematic review of the literature. *Patient Relat Outcome Meas.* 2015;6:19-51.
9. Stone DS, Ganz PA, Pavlish C, Robbins WA. Young adult cancer survivors and work: a systematic review. *J Cancer Surviv.* 2017;11(6):765-781.
10. Spathis A, Hatcher H, Booth S, et al. Cancer-related fatigue in adolescents and young adults after cancer treatment: persistent and poorly managed. *J Adolesc Young Adult Oncol.* 2017;6(3):489-493.
11. Richter D, Koehler M, Friedrich M, Hilgendorf I, Mehnert A, Weissflog G. Psychosocial interventions for adolescents and young adult cancer patients: A systematic review and meta-analysis. *Crit Rev Oncol Hematol.* 2015;95(3):370-386.
12. Brunet J, Wurz A, Shallwani SM. A scoping review of studies exploring physical activity among adolescents and young adults diagnosed with cancer. *Psychooncology.* 2018;27(8):1875-1888.
13. Pugh G, Gravestock HL, Hough RE, King WM, Wardle J, Fisher A. Health behavior change interventions for teenage and young adult cancer survivors: a systematic review. *J Adolesc Young Adult Oncol.* 2016;5(2):91-105.
14. Frazelle ML, Friend PJ. Optimizing the teachable moment for health promotion for cancer survivors and their families. *J Adv Pract Oncol.* 2016;7(4):422-433.
15. Baumann FT, Bloch W, Beulertz J. Clinical exercise interventions in pediatric oncology: a systematic review. *Pediatr Res.* 2013;74(4):366-374.
16. Mishra SI, Scherer RW, Geigle PM, et al. Exercise interventions on health-related quality of life for cancer survivors. *Cochrane Database Syst Rev.* 2012;8:CD007566.
17. Albrecht TA, Taylor AG. Physical activity in patients with advanced-stage cancer: a systematic review of the literature. *Clin J Oncol Nurs.* 2012;16(3):293-300.
18. Wurz A, Brunet, J. The effects of physical activity on health and quality of life in adolescent cancer survivors: a systematic review. *JMIR Cancer.* 2016;2(1):e6.

19. Rock CL, Doyle C, Demark-Wahnefried W, et al. Nutrition and physical activity guidelines for cancer survivors. *CA Cancer J Clin.* 2012;62(4):243-274.
20. Schmitz KH, Courneya KS, Matthews C, et al. American College of Sports Medicine roundtable on exercise guidelines for cancer survivors. *Med Sci Sports Exerc.* 2010;42(7):1409-1426.
21. Chamorro-Vina C, Keats M, Culos-Reed SN. *Pediatric Oncology Exercise Manual.* Calgary, AB: Health & Wellness Lab, University of Calgary;2015.
22. Murnane A, Gough K, Thompson K, Holland L, Conyers R. Adolescents and young adult cancer survivors: exercise habits, quality of life and physical activity preferences. *Support Care Cancer.* 2015;23(2):501-510.
23. Thomas DM, Albritton KH, Ferrari A. Adolescent and young adult oncology: an emerging field. *J Clin Oncol.* 2010;28(32):4781-4782.
24. Milosevic E, Brunet J, Campbell K. Exploring tensions within young breast cancer survivors' physical activity, nutrition and weight management beliefs and practices. *Disability Rehab.* Accepted.
25. Pugh G, Hough RE, Gravestock HL, Jackson SE, Fisher A. The health behavior information needs and preferences of teenage and young adult cancer survivors. *J Adolesc Young Adult Oncol.* 2017;6(2):318-326.
26. Rabin C. Barriers to increasing physical activity among young adult cancer survivors. *J Adolesc Young Adult Oncol.* 2017;6(2):372-376.
27. Rabin C, Simpson N, Morrow K, Pinto B. Behavioral and psychosocial program needs of young adult cancer survivors. *Qual Health Res.* 2011;21(6):796-806.
28. Zebrack B. Information and service needs for young adult cancer survivors. *Support Care Cancer.* 2009;17(4):349-357.
29. Zebrack BJ, Mills J, Weitzman TS. Health and supportive care needs of young adult cancer patients and survivors. *J Cancer Surviv.* 2007;1(2):137-145.
30. Michie S JM, Francis J, Hardeman W, Eccles M. From theory to intervention: mapping theoretically derived behavioural determinants to behaviour change techniques. *Appl Psychol.* 2008;57:660-680.
31. Deci EL, Ryan, R.M. *Handbook of self-determination research.* Rochester, NY: University of Rochester Press;2002.
32. Teixeira PJ, Carraca EV, Markland D, Silva MN, Ryan RM. Exercise, physical activity, and self-determination theory: a systematic review. *Int J Behav Nutr Phys Act.* 2012;9:78.
33. Silva MN, Marques MM, Teixeira PJ. Testing theory in practice: The example of self-determination theory-based interventions. *Eur Health Psychol.* 2014;16(5):171-180.
34. Teixeira PJ, Carraca EV, Marques MM, et al. Successful behavior change in obesity interventions in adults: a systematic review of self-regulation mediators. *BMC Med.* 2015;13:84.
35. Deci EL, Ryan, R.M. A motivational approach to self: integration in personality. *Neb Symp Mot.* 1990;38:237-288.
36. Edmunds JK, Duda JL, Ntoumanis N. Psychological needs and the prediction of exercise-related cognitions and affect among an ethnically diverse cohort of adult women. *Int J Sport Exerc Psychol.* 2010;8:446-463.
37. Milne HM, Wallman KE, Guilfoyle A, Gordon S, Corneya KS. Self-determination theory and physical activity among breast cancer survivors. *J Sport Exerc Psychol.* 2008;30(1):23-38.

38. Peddle CJ, Plotnikoff RC, Wild TC, Au HJ, Courneya KS. Medical, demographic, and psychosocial correlates of exercise in colorectal cancer survivors: an application of self-determination theory. *Support Care Cancer.* 2008;16(1):9-17.
39. Wilson PM, Blanchard CM, Nehl E, Baker F. Predicting physical activity and outcome expectations in cancer survivors: an application of self-determination theory. *Psychooncology.* 2006;15(7):567-578.
40. Gillison F SM, Rouse P, Sebire SJ, Ryan R. A meta-analysis of the efficacy of techniques to promote the internalization of motivation for health-related behaviors: a self-determination theory perspective. *Health Psych Rev.* 2018;epub ahead of print.
41. Hallward L, Patel N, Duncan LR. Behaviour change techniques in physical activity interventions for men with prostate cancer: a systematic review. *J Health Psychol.* 2018:1359105318756501.
42. Stacey FG, James EL, Chapman K, Courneya KS, Lubans DR. A systematic review and meta-analysis of social cognitive theory-based physical activity and/or nutrition behavior change interventions for cancer survivors. *J Cancer Surviv.* 2015;9(2):305-338.
43. Bluethmann SM, Vernon SW, Gabriel KP, Murphy CC, Bartholomew LK. Taking the next step: a systematic review and meta-analysis of physical activity and behavior change interventions in recent post-treatment breast cancer survivors. *Breast Cancer Res Treat.* 2015;149(2):331-342.
44. Sylvester BD, Zammit K, Fong AJ, Sabiston CM. An evaluation of the behaviour-change techniques used on Canadian cancer centre websites to support physical activity behaviour for breast cancer survivors. *Curr Oncol.* 2017;24(6):e477-e485.
45. Rabin C, Simpson N, Morrow K, Pinto B. Intervention format and delivery preferences among young adult cancer survivors. *Int J Behav Med.* 2013;20(2):304-310.
46. Love B, Crook B, Thompson CM, et al. Exploring psychosocial support online: a content analysis of messages in an adolescent and young adult cancer community. *Cyberpsychol Behav Soc Netw.* 2012;15(10):555-559.
47. Mooney R, Samhouri M, Holton A, et al. Adolescent and young adult cancer survivors' perspectives on their Internet use for seeking information on healthy eating and exercise. *J Adolesc Young Adult Oncol.* 2017;6(2):367-371.
48. Chou WY, Liu B, Post S, Hesse B. Health-related Internet use among cancer survivors: data from the Health Information National Trends Survey, 2003-2008. *J Cancer Surviv.* 2011;5(3):263-270.
49. Dolce MC. The Internet as a source of health information: experiences of cancer survivors and caregivers with healthcare providers. *Oncol Nurs Forum.* 2011;38(3):353-359.
50. Kenten C, Martins A, Fern LA, et al. Qualitative study to understand the barriers to recruiting young people with cancer to BRIGHTLIGHT: a national cohort study in England. *BMJ Open.* 2017;7(11):e018291.
51. Friend BD, Baweja A, Schiller G, et al. Clinical trial enrollment of adolescent and young adult patients with cancer: a systematic review of the literature and proposed solutions. *Clin Onc Adol Young Adult.* 2016;6.
52. Schnur JB, Montgomery GH. E-counseling in psychosocial cancer care: a survey of practice, attitudes, and training among providers. *Telemed J E Health.* 2012;18(4):305- 308.

53. Norman GJ, Zabinski MF, Adams MA, Rosenberg DE, Yaroch AL, Atienza AA. A review of eHealth interventions for physical activity and dietary behavior change. *Am J Prev Med.* 2007;33(4):336-345.
54. Muellmann S, Forberger S, Mollers T, Zeeb H, Pischke CR. Effectiveness of eHealth interventions for the promotion of physical activity in older adults: a systematic review protocol. *Syst Rev.* 2016;5:47.
55. Aalbers T, Baars MA, Rikkert MG. Characteristics of effective Internet-mediated interventions to change lifestyle in people aged 50 and older: a systematic review. *Age Res Rev.* 2011;10(4):487-497. Davies CA, Spence JC, Vandelanotte C, Caperchione CM, Mummery WK. Meta- analysis of Internet-delivered interventions to increase physical activity levels. *Int J Behav Nutr Phys Act.* 2012;9:52.
56. Foster C, Richards J, Thorogood M, Hillsdon M. Remote and web 2.0 interventions for promoting physical activity. *Cochrane Database Syst Rev.* 2013;9:CD010395.
57. Krebs P, Prochaska JO, Rossi JS. A meta-analysis of computer-tailored interventions for health behavior change. *Prev Med.* 2010;51(3-4):214-221.
58. World Health Organization. *E-Health.* World Health Organization, Geneva;2015.
59. Czajkowski SM, Powell LH, Adler N, et al. From ideas to efficacy: The ORBIT model for developing behavioral treatments for chronic diseases. *Health Psychol.* 2015;34(10):971-982.
60. Craig P, Dieppe P, Macintyre S, et al. Developing and evaluating complex interventions: the new Medical Research Council guidance. *BMJ.* 2008;337:a1655.
61. American College of Sports Medicine. *Exercising with cancer.* American College of Sports Medicine. 2018: https://www.cancer.org/treatment/survivorship-during-and-after- treatment/staying-active/physical-activity-and-the-cancer-patient.html.
62. O'Dwyer T, Monaghan A, Moran J, O'Shea F, Wilson F. Behaviour change intervention increases physical activity, spinal mobility and quality of life in adults with ankylosing spondylitis: a randomised trial. *J Physiother.* 2017;63(1):30-39.
63. Howlett N, Trivedi D, Troop NA, Chater AM. Are physical activity interventions for healthy inactive adults effective in promoting behavior change and maintenance, and which behavior change techniques are effective? A systematic review and meta- analysis. *Transl Behav Med.* 2018.
64. Thabane L, Ma J, Chu R, et al. A tutorial on pilot studies: the what, why and how. *BMC Med Res Methodol.* 2010;10:1.
65. Lancaster GA, Dodd S, Williamson PR. Design and analysis of pilot studies: recommendations for good practice. *J Eval Clin Pract.* 2004;10(2):307-312.
66. Whitehead AL, Julious SA, Cooper CL, Campbell MJ. Estimating the sample size for a pilot randomised trial to minimise the overall trial sample size for the external pilot and main trial for a continuous outcome variable. *Stat Methods Med Res.* 2016;25(3):1057- 1073.
67. Wurz A, Brunet, J. Exploring the feasibility, safety, and potential benefits of a 12-week home-based physical activity intervention. 2017;NCT03016728:https://clinicaltrials.gov/ct2/show/NCT03016728.
68. Schulz KF, Grimes DA. Sample size slippages in randomised trials: exclusions and the lost and wayward. *Lancet.* 2002;359(9308):781-785.
69. Valle CG, Tate DF, Mayer DK, Allicock M, Cai J. A randomized trial of a Facebook-based physical activity intervention for young adult cancer survivors. *J Canc Surviv.* 2013;7(3):355-368.

70. Rabin C, Dunsiger S, Ness KK, Marcus BH. Internet-based physical activity intervention targeting young adult cancer survivors. *J Adolesc Young Adult Oncol.* 2011;1(4):188- 194.
71. Rabin C, Horowitz S, Marcus B. Recruiting young adult cancer survivors for behavioral research. *J Clin Psychol Med Settings.* 2013;20(1):33-36.
72. Van der Gucht K, Takano K, Labarque V, et al. A Mindfulness-based intervention for adolescents and young adults after cancer treatment: effects on quality of life, emotional distress, and cognitive vulnerability. *J Adolesc Young Adult Oncol.* 2017;6(2):307-317.
73. Broderick JM, Ryan J, O'Donnell DM, Hussey J. A guide to assessing physical activity using accelerometry in cancer patients. *Support Care Cancer.* 2014;22(4):1121-1130.
74. McClain JJ, Sisson SB, Tudor-Locke C. Actigraph accelerometer inter-instrument reliability during free-living in adults. *Med Sci Sports Exerc.* 2007;39(9):1509-1514.
75. Plasqui G, Westerterp KR. Physical activity assessment with accelerometers: an evaluation against doubly labeled water. *Obesity.* 2007;15(10):2371-2379.
76. Robusto KM, Trost SG. Comparison of three generations of ActiGraph activity monitors in children and adolescents. *J Sports Sci.* 2012;30(13):1429-1435.
77. Santos-Lozano A, Marin PJ, Torres-Luque G, Ruiz JR, Lucia A, Garatachea N. Technical variability of the GT3X accelerometer. *Med Eng Phys.* 2012;34(6):787-790.
78. Ware JE, Jr., Sherbourne CD. The MOS 36-item short-form health survey (SF-36). I. Conceptual framework and item selection. *Med Care.* 1992;30(6):473-483.
79. Ware J, Kosinski, M., Bjorner, J., Turner-Bowker, D., Gandek, B., Maruish, M. *Development. User's Manual for the SF-36v2® Health Survey.* Lincoln (RI): Quality Metric Incorporated;2007.
80. McHorney CA, Ware JE, Jr., Lu JF, Sherbourne CD. The MOS 36-item Short-Form Health Survey (SF-36): III. Tests of data quality, scaling assumptions, and reliability across diverse patient groups. *Med Care.* 1994;32(1):40-66.
81. Watson, D., Clark, L. A., & Tellegen, A. (1988). Development and validation of brief measures of positive and negative affect: the PANAS scales. *Journal of personality and social psychology*, *54*(6), 1063.
82. Kroenke K, Spitzer RL, Williams JBW. The PHQ-9: Validity of a brief depression severity measure. J Gen Intern Med 2001;16:606-613.
83. Spitzer RL, Kroenke K, Williams JBW, Löwe B. A brief measure for assessing generalized anxiety disorder: the GAD-7. Arch Intern Med 2006;166:1092-1097.
84. Husson O, Zebrack BJ. Psychometric evaluation of an adolescent and young adult module of the impact of cancer instrument. *J Adolesc Young Adult Oncol.* 2017;6(1):159-170.
85. Vander Zee KI, Sanderman R, Heyink JW, de Haes H. Psychometric qualities of the RAND 36-Item Health Survey 1.0: a multidimensional measure of general health status. *Int J Behav Med.* 1996;3(2):104-122.
86. Brunet J, Burke S, Grocott MP, West MA, Jack S. The effects of exercise on pain, fatigue, insomnia, and health perceptions in patients with operable advanced stage rectal cancer prior to surgery: a pilot trial. *BMC Cancer.* 2017;17(1):153.
87. Hughes S, Jaremka LM, Alfano CM, et al. Social support predicts inflammation, pain, and depressive symptoms: longitudinal relationships among breast cancer survivors. *Psychoneuroendocrinology.* 2014;42:38-44.
88. Cary KC, Singla N, Cowan JE, Carroll PR, Cooperberg MR. Impact of androgen deprivation therapy on mental and emotional well-being in men with prostate cancer: analysis from the CaPSURE registry. *J Urol.* 2014;191(4):964-970.

89. Heins MJ, Korevaar JC, Hopman PE, Donker GA, Schellevis FG, Rijken MPM. Health-related quality of life and health care use in cancer survivors compared with patients with chronic diseases. *Cancer.* 2016;122:196-170.
90. Ell K, Sanchez K, Vourlekis B, et al. Depression, correlates of depression, and receipt of depression care among low-income women with breast or gynecological cancer. *J Clin Oncol.* 2005;23(13):3052-3060.
91. Gorman J, Su I, Pierce J, et al. A multidimensional scale to measyre rge reproductive converns of young adult female cancer survivors. *J Cancer Surviv.* 2014;8(2):218-228.
92. Brown L, Kroenke K, Theobald D, et al. The association of depression and anxiety with health-related quality of life in cancer patients with depression and/or pain. *Psycho-Onc.* 2010;19:734-741.
93. Danhauer SC, Rutherford CA, Hurt G, Gentry S, Lovato J, McQuellon RP. Providing psychosocial group support for young women with breast cancer: findings from a wellness-based community collaboration. *J Psychosoc Oncol.* 2007;25(1):103-120.
94. Spitzer RL, Kroenke K, Williams JBW, Lowe B. A brief measure for assessing generalized anxiety disorder. *Arch Intern Med*. 2006;166:1092–1097.
95. Thekkumpurath P, Walker J, Butcher I, et al. Screening for major depression in cancer outpatients: the diagnosric accuracy of the 9-item patient health questionnaire. *Cancer.* 2010;117(1):218-27.
96. Michie S. Designing and implementing behaviour change interventions to improve population health. *J Health Serv Res Policy.* 2008;13 Suppl 3:64-69.
97. Sniehotta FF. Towards a theory of intentional behaviour change: plans, planning, and self-regulation. *Br J Health Psychol.* 2009;14(Pt 2):261-273.
98. Markland D, Tobin VA. Modification of the Behavioral Regulation in Exercise Questionnaire to include an assessment of amotivation. *Journal of Sport and Exercise Psychology,.* 2004;26:191096.
99. Wilson PM, Rodgers, W.M., Loitz, C.C., Scime, G. “It’s who I am...really!” The importance of integrated regulation in exercise contexts. *Journal of Biobehavioral Research.* 2006;11:79-104.
100. Wilson PM, Rogers WT, Rodgers WM, Wild TC. The Psychological Need Satisfaction in Exercise Scale. *J Sport Exerc Psychol.* 2006;28(3):231-251.
101. Gunnell KE, Wilson PM, Zumbo BD, Mack DE, Crocker PRE. Assessing psychological need satisfaction in exercise contexts: issues of score invariance, item modification, and context. *Measure Phys Ed Exerc Sci.* 2012;16(3):219-236.
102. Williams GC, Grow VM, Freedman ZR, Ryan RM, Deci EL. Motivational predictors of weight loss and weight-loss maintenance. *J Pers Soc Psychol.* 1996;70(1):115-126.
103. Marcus BH, Rakowski W, Rossi JS. Assessing motivational readiness and decision making for exercise. *Health Psychol.* 1992;11(4):257-261.
104. Marcus BH, Rossi JS, Selby VC, Niaura RS, Abrams DB. The stages and processes of exercise adoption and maintenance in a worksite sample. *Health Psychol.* 1992;11(6):386-395.
105. Sallis JF, Grossman RM, Pinski RB, Patterson TL, Nader PR. The development of scales to measure social support for diet and exercise behaviors. *Prev Med.* 1987;16(6):825-836.
106. Linn BS LM, Gurel L. Cumulative illness rating scale. *J Am G Soc.* 1968;16(5).

107. Neuendorf KA. *The Content Analysis Guidebook.* 2 ed. Thousand Oaks, CA: Sage Publications;2017.

108. Hooper R, Forbes A, Hemming K, Takeda A, Beresford L. Analysis of cluster randomised trials with an assessment of outcome at baseline. *BMJ.* 2018;360:k1121.

109. Fitzmaurice G, Laird NJW. *Applied Longitudinal Analysis.* 2 ed: John Wiley & Sons;2011.

110. Lee EC, Whitehead AL, Jacques RM, Julious SA. The statistical interpretation of pilot trials: should significance thresholds be reconsidered? *BMC Med Res Methodol.* 2014;14:41.
